# Surprise Acts as a Reducer of Outcome Value in Human Reinforcement Learning

**DOI:** 10.3389/fnins.2020.00852

**Published:** 2020-09-08

**Authors:** Motofumi Sumiya, Kentaro Katahira

**Affiliations:** ^1^Department of Cognitive and Psychological Sciences, Graduate School of Informatics, Nagoya University, Nagoya, Japan; ^2^Japan Society for the Promotion of Science, Tokyo, Japan

**Keywords:** surprise, reward prediction error, reinforcement learning, risk, decision making, outcome value

## Abstract

Surprise occurs because of differences between a decision outcome and its predicted outcome (prediction error), regardless of whether the error is positive or negative. It has recently been postulated that surprise affects the reward value of the action outcome; studies have indicated that increasing surprise as an absolute value of prediction error decreases the value of the outcome. However, how surprise affects the value of the outcome and subsequent decision making is unclear. We suggest that, on the assumption that surprise decreases the outcome value, agents will increase their risk-averse choices when an outcome is often surprising. Here, we propose the surprise-sensitive utility model, a reinforcement learning model that states that surprise decreases the outcome value, to explain how surprise affects subsequent decision making. To investigate the properties of the proposed model, we compare the model with previous reinforcement learning models on two probabilistic learning tasks by simulations. As a result, the proposed model explains the risk-averse choices like the previous models, and the risk-averse choices increase as the surprise-based modulation parameter of outcome value increases. We also performed statistical model selection by using two experimental datasets with different tasks. The proposed model fits these datasets better than the other models with the same number of free parameters, indicating that the model can better capture the trial-by-trial dynamics of choice behavior.

## Introduction

Decision making in everyday life depends on predicting outcomes associated with potential choices. The prediction is updated by the actual outcome obtained after the choice. These processes can be modeled by a reinforcement learning algorithm, a framework that uses prediction error as a learning signal to update future outcome expectations (Schultz, 2016). Prediction error represents the difference between actual and expected outcomes and has a positive or negative valence. When a decision outcome exceeds expectations, the value associated with the chosen option is increased, making it more likely to be chosen again. When a decision outcome is less than expected, the value associated with the chosen option is decreased, making it less likely to be chosen again. Prediction error also functions as a surprise, based on the degree of absolute prediction error. When people are confronted with unexpected events, regardless of their valence, they experience surprise. A greater surprise increases the degree of the change in the expectation (Niv et al., 2015; Haarsma et al., 2019). However, how surprise affects the value of the outcome and subsequent decision making remains unclear.

Recent experimental human and monkey studies have shown that surprising events or outcomes harm individuals, even when the error is positive (Knight et al., 2013; Topolinski and Strack, 2015). Topolinski and Strack (2015) used facial electromyography to measure people’s responses immediately after surprising information; they observed that participants flexed their corrugator muscles, which indicated negative valence. Knight et al. (2013) showed that in a predictable food-taking paradigm, rhesus monkeys were slower to accept unexpected offers and exhibited aversive reactions, such as repeatedly turning their heads and looking away before accepting the food item, especially in response to better-than-expected offers. These studies indicate that surprise decreases, at least initially, the value of an event for individuals and decreases the value of the offer associated with the event.

This phenomenon, reducing the outcome value by surprise, can be indicated from the perspective of predictive coding, which is a computational theory of brain function. This theory postulates that the brain facilitates perception from sensation by minimizing the prediction error between the expected and received sensory input (Friston, 2005; Clark, 2013). Some researchers using this theory have proposed that surprise affects the reward value of a decision outcome (Peters et al., 2017; Van de Cruys, 2017; Friston, 2018). They have stated that an increase in surprise decreases the value of the outcome, as surprise makes a situation uncertain, which is generally associated with negative affect. For example, Van de Cruys (2017) suggested that positive affect is induced when prediction error is reduced, whereas negative affect is induced when a situation with lower prediction errors shifts to one with higher prediction errors.

Here, we propose the surprise-sensitive utility model, in which the degree of outcome surprise affects the outcome value. We investigated how the effect of surprise on the outcome value affects subsequent decisions by using two reinforcement learning tasks: the risky, probabilistic learning task (Niv et al., 2012) and the simple two-armed bandit task. We predicted that a surprising outcome that decreases the value of a decision outcome would decrease the preference for risky choices, in which prediction error often occurs. To investigate this prediction, we first simulated the risk-sensitive learning task and compared the proposed model with previous computational models on the task. We also fitted the models to real experimental data taken from Niv et al. (2012). We found a better fit of the proposed model to the dataset than previous models and conducted model selection using an additional dataset (Goris et al., 2019) to investigate the generalizability of the proposed model to different tasks and populations. Furthermore, we simulated the simple two-armed bandit task to investigate how parameters within the proposed model modulate the choice preference.

### Model Description

The Q-learning model is the base model of our surprise-sensitive utility model. The Q-learning model incorporates a Rescorla–Wagner rule, where only the Q-value of the chosen option is updated by a prediction error, which explains the observed behavior by computing an action value *Q*(*t*) for each trial *t*, which represents the expected outcome of the action. The *Q*-value of the chosen action is updated iteratively by a prediction error, which is the difference between the expected outcome *Q*(*t*) and the received outcome *r*. Based on Niv et al. (2012), a decaying term of 0.5/(1+*T*_*s*_) (with *T*_*s*_ being the number of trials in which the consequences of stimulus S had been experienced) was added to a constant learning rate, α as follows: α′ = α + 0.5/(1+*T*_*s*_)

$$Q\,\left(t+1\right)=Q\,\left(t\right)+\alpha^{{}^{\prime }}\,\left(r\,\left(t\right)-Q\,\left(t\right)\right)$$

The surprise-sensitive utility model is a model in which the received outcome *r* is affected by the surprise (absolute value of prediction error). In this model, *S*(*t*) is the subjective utility modulated by surprise. The degree of modulation is controlled by a parameter *d* as follows:

S⁢(t)=r⁢(t)-d⁢|r⁢(t)-Q⁢(t)|

$$Q\,\left(t+1\right)=Q\,\left(t\right)+\alpha^{{}^{\prime }}\,\left(S\,\left(t\right)-Q\,\left(t\right)\right)$$

For all models, the probability of choosing option *i* at trial *t* is given by the softmax function:

$$P\left(a\left(t\right)=i\right)=\frac{e\,x\,p\,\left(\beta \cdot Q_{i}\,\left(t\right)\right)}{\sum_{j=1}^{K}e\,x\,p\,\left(\beta \cdot Q_{j}\,\left(t\right)\right)}$$

where β is the inverse temperature parameter that determines the sensitivity of the choice probabilities to differences in the values, and *K* represents the number of possible actions. In this study, *K* = 2 unless otherwise stated. *a*(*t*) denotes the option that was chosen at trial *t*.

## Materials and Methods

### Analysis 1: Simulation With a Risk-Sensitive Task

We first simulated a risk-sensitive task with 1,000 agents and compared the probability of choosing a sure option on four models: the proposed model (the surprise-sensitive utility model) and three previous models described in Niv et al. (2012) (the Q-learning model, utility model, and risk-sensitive Q-learning model). The utility model is a Q-learning model that incorporates non-linear subjective utilities for the different reward amounts. In this model, *U*(*x*) indicates the subjective utility of a reward whose objective value is *x*: *U*(0) =  0, *U*(20) =  20, and *U*(40) = *a*⋅20. The value of *a* affects the utility curve. The risk-sensitive model is a Q-learning model in which positive and negative prediction errors have asymmetric effects on learning. Specifically, there are separate learning rates: α^+^ and α^−^ for positive and negative prediction errors, respectively.

To show the similarity of the individual-level probabilities of a sure choice estimated from each model and real data, we simulated the choice behavior for every 16 subjects for each model. For the parameter values of each model (α and β for the Q-learning model; α, β, and u for the utility model; α^+^, α^−^, and β for the risk-sensitive model; α, β, and *d* for the surprise model), we used the median of the best-fitting parameter values from 16 participants’ data in Niv et al. (2012) (see *Analysis 2* for details of model fitting). To make the model performance clearer, we simulated the task (234 trials) 1,000 times and calculated the mean of the probability of choosing a sure option for each subject.

Furthermore, within the surprise-sensitive utility model, we examined how the free parameters α and *d* are related to the risk-sensitive choice. For the surprise decay rate *d*, we ran simulations for 1,000 times with each of three learning rates (α = 0.2, 0.5, and 0.8).

### Risky, Probabilistic Learning Task

The risky, probabilistic learning task is described in detail in Niv et al. (2012). Briefly, five different-colored stimuli (portrayed as casino-style slot machines or bandits) were randomly allocated to five payoff schedules: sure 40/c, sure 20/c, two sure 0/c stimuli, and one variable-payoff risky stimulus associated with equal probabilities of 0/c or 40/c payoffs. Two types of trials were intermixed randomly: choice trials and forced trials. In choice trials, two stimuli were displayed, and the subject was instructed to select one of them quickly. In forced trials, only one stimulus was displayed, and the subject was forced to select it and obtain its associated outcome. The task consisted of 234 trials (three sessions of 78 trials). Each trial comprised (1) 30 “risk” choice trials involving a choice between the 20/c stimulus and the 0/40/c stimulus (target choice to assess subjects’ behavioral risk sensitivity); (2) 20 “test” choice trials involving each of the pairs 40/c vs. 0/40/c, 20/c vs. 40/c, 0/c vs. 0/40/c, and 0/c vs. 20/c (3) 24 forced trials involving each of the stimuli (16 only for each of the 0/c stimuli); and (4) 20 trials in which subjects chose between the two 0/c stimuli.

### Analysis 2-1: Model Selection With Actual Experimental Data

To investigate how the model fits the actual experimental data, we conducted a model comparison based on the actual experimental data from Niv et al. (2012) presented on the author’s homepage^1^. This dataset consists of data from 16 participants who conducted the same risk-sensitive task as the simulation.

The free parameters of each model for model fitting were as follows: α and β for the Q-learning model; α, β, and u for the utility model; α^+^, α^−^, and β for the risk-sensitive model; α, β, and *d* for the surprise model (Supplementary Table S1). The learning rate was constrained to the range 0≤α,α^+^,α^−^≤1 with a beta (2,2) prior distribution, and the inverse temperature was constrained to 0≤β≤10 with a gamma (2,3) prior distribution. The utility parameter was constrained to the range 1≤*a*≤30 with a uniform prior distribution; these priors are based on Niv et al. (2012). Additionally, the decay rate of the surprise model was constrained to −1≤*d*≤1 with a uniform prior distribution. To test whether the value of d was positive, we allowed this parameter to be negative. We fit the parameters of each model with a maximum *a posteriori* estimation. We also approximated the log marginal likelihood (evidence) for each model by using the Laplace approximation (Kass and Raftery, 1995). We used the R function “solnp” in the Rsolnp package (Ghalanos and Theussl, 2015) to estimate the fitting parameters. We confirmed whether the estimated surprise decay parameter was positive or negative using a one-sample *T*-test. In the model selection, model evidence (log marginal likelihood) for each model and participant was subjected to random-effects Bayesian model selection (BMS; Stephan et al., 2009) by using the function “spm_BMS” in SPM12. BMS provides estimates of the protected exceedance probability, defined as the probability that a particular model is more frequent in the population among the set of candidate models.

### Analysis 2-2: Model Selection With Actual Experimental Data

To ensure the fitting of the proposed model in different tasks and populations, we also conducted model selection with the actual experimental datasets from Goris et al. (2019) whose datasets are presented on the Open Science Framework website^2^. The datasets include the autism spectrum condition scores for each participant. However, we did not consider the participants’ autistic score. This is because the purpose of this analysis was to confirm the fitness of the newly proposed model among other models but not to investigate the specific characteristics of psychiatric disorders, which will be done in future studies.

Here, we briefly describe their task and utilized computational models. The Goris et al. (2019) dataset consists of data from 163 participants who conducted a gambling task, which is a kind of four-armed bandit task, with 200 trials. Each of the four choices had the same average reward outcome, € 250, but different predictability. These choices returned a gain drawn from a normal distribution with a mean of € 250 and a standard deviation of € 0, € 10, € 30, and € 70. We fitted the models from Niv et al. (2012) and the proposed model to the data. The models and the methods of model selection were the same as the Niv et al. (2012) dataset but with different numbers of possible actions (*K* = 4).

### Analysis 3: Simulation With Simple Two-Armed Bandit Tasks

To investigate the behavioral difference between models on a simple task, we simulated the simple two-armed bandit task and investigated the relationship between stay probability and reward history. We simulated 1,000 agents using the surprise-sensitive utility model and the basic Q-learning model. We compared the stay probability, defined as the probability of choosing the same choice in the next trial, *t+1*, as the current trial, *t*. In the task, agents chose one of two choices in which the probability of reward acquisition was different (0.8 vs. 0.2). The parameter values for each model were α = 0.3 and β = 2 for Q-learning and α = 0.3, β = 2, and *d* = 0.5 for the surprise model. Furthermore, we investigated the influence of the surprise decay rate (*d*) on the stay probability by varying the value of *d* as *d* = 0.1, 0.5, and 0.9 within the surprise-sensitive utility model. We predicted that the stay probability of the proximate reward condition [*r*(*t*) = 1] would decrease as the surprise decay rate increased.

To explore the detail in the difference between the surprise-sensitive utility model and the basic Q-learning model, we conducted two additional simulations with simple bandit tasks whose two options were different on probability or outcome value. Specifically, we simulated the task with two safe options (reward probability: *p* = 0.9) with a small value difference (*r* = 1 or 0.8) and the task with two safe options (p = 0.9) associated with a higher value (*r* = 1) or lower value (*r* = 0.3).

## Results

**FIGURE 1:**
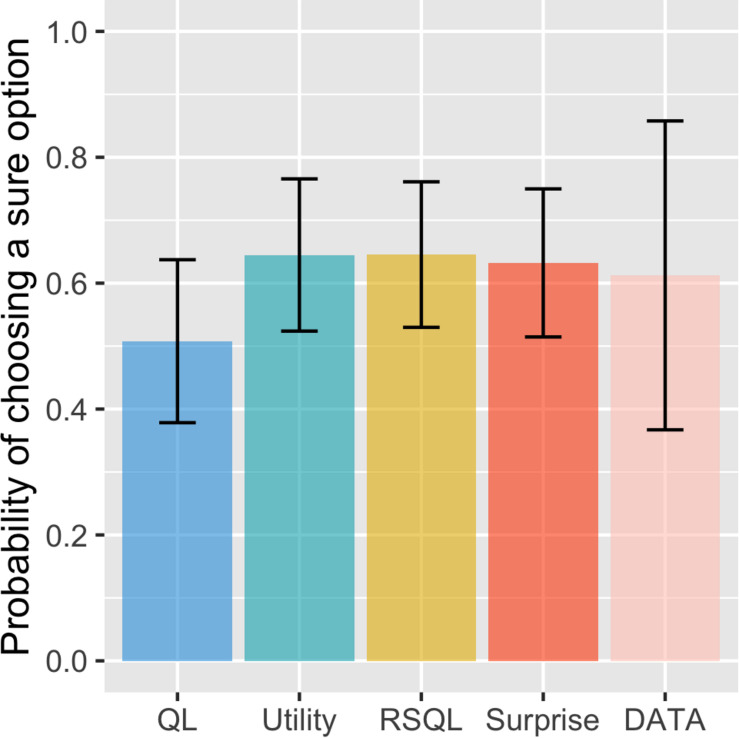
Probability of choosing a sure option for model and real data. Data are presented as the mean ± standard error. The surprise-sensitive utility model had a similar probability of choosing a sure option (risk aversion) as the risk-sensitive Q-learning model and the utility model. Additionally, the probability of choosing a sure option simulated with the proposed model was similar to the result of real experimental data. QL, Q-learning model; utility, utility model; RSQL, risk-sensitive Q-learning model; surprise, surprise-sensitive utility model.

We first performed the simulation to investigate the risk aversion tendency for the four models. The previous winning model was the risk-sensitive Q-learning model, which had separate learning rates in which positive and negative prediction errors have asymmetric effects on learning. As shown in Figure 1, the surprise-sensitive utility model had a similar probability of choosing a sure option (i.e., risk aversion) as the risk-sensitive Q-learning model and the utility model. Additionally, the probability of choosing a sure option simulated with the proposed model was similar to the result of actual experimental data from Niv et al. (2012). Furthermore, we found that the probabilities of sure choice of the proposed model fitted to individual subjects were more similar to the real data than other models for some subjects, especially whose probability of choosing a sure option was high (Supplementary Figure S1). Compared with the other models, the proposed model showed the most similar probability of choosing a sure option on the top four subjects choosing the sure choice. This finding indicates that the proposed model may better explain the choice behavior of risk-averse agents.

**FIGURE 2:**
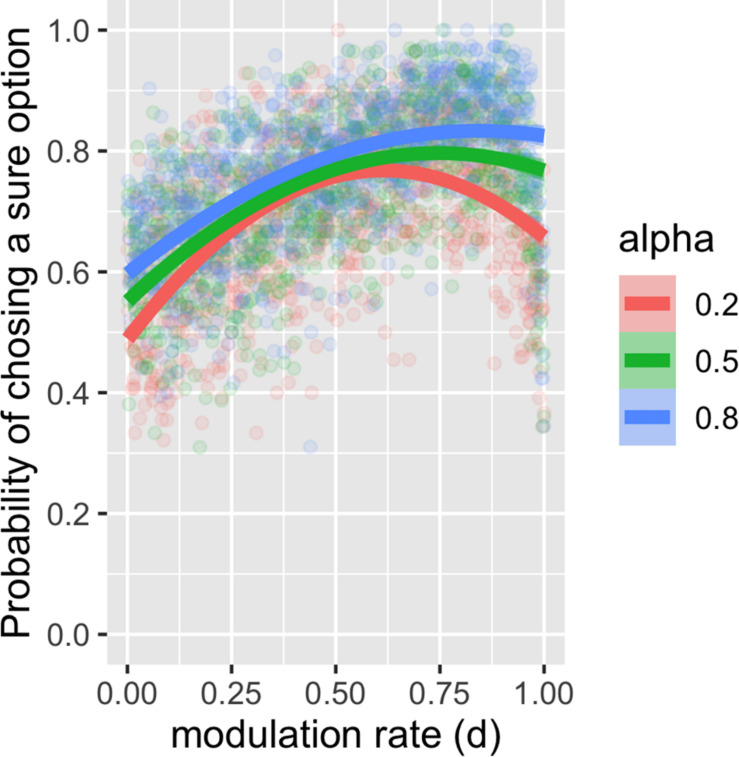
Plot and second-order polynomial fit for the probability of choosing a sure option depending on the surprise decay rate and learning rate. We found that risk aversion increased as the surprise decay rate (*d*) increased in the surprise-sensitive utility model and that this tendency increased as the learning rate (α) increased.

Furthermore, within the surprise-sensitive utility model, we examined how the learning rate α and the surprise decay rate *d* are related to the risk-sensitive choice. We found that risk aversion increased as *d* increased in the surprise-sensitive utility model (Figure 2) and that this tendency was more pronounced as α increased. For Figure 2, we restricted the value of *d* from 0 to 1, as this study focused on the reduction in outcome value by surprise (see Supplementary Figure S2 for the case with a negative *d* value).

**FIGURE 3:**
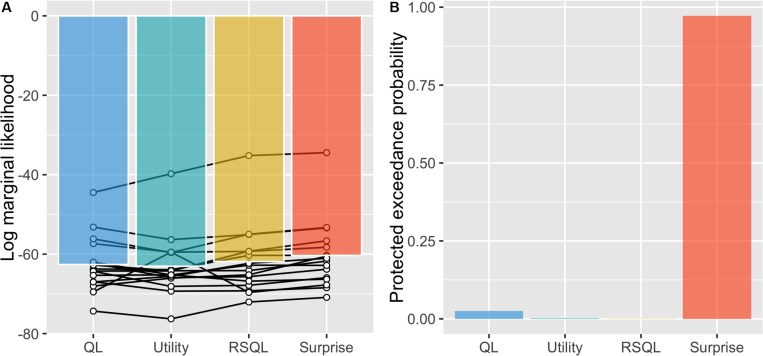
Model selections. **(A)** We compared the model evidence of each model (log marginal likelihood) and found that the surprise-sensitive utility model had the largest value. **(B)** We carried out a Bayesian model comparison and found that the surprise-sensitive utility model had a decisively higher protected exceedance probability. QL, Q-learning model; utility, utility model; RSQL, risk-sensitive Q-learning model; surprise, surprise-sensitive utility model.

We next conducted model selection with actual experimental data of the risky, probabilistic learning task from Niv et al. (2012). We compared the model evidence of each model (log marginal likelihood) and found that the surprise-sensitive utility model had the largest value (Figure 3A). We then carried out a Bayesian model comparison to determine the best model for explaining choice behavior and found that the surprise-sensitive utility model had a decisively higher protected exceedance probability, indicating that it is more frequent in the population than the other models (Figure 3B). Each estimated parameter of the model is shown in Supplementary Figure S3. We confirmed that the estimated surprise decay parameter was significantly positive even with a uniform prior distribution from -1 to 1 [*t*(15) = 9.34, *p* < 0.0001]. These findings indicate that modeling the diminishing effect of surprise on outcome values led to a better fit to the dataset than the other models.

To investigate the generalizability of the proposed model to different tasks and populations, we performed model selection using a dataset from Goris et al. (2019). In this dataset, the subjects performed a four-armed bandit task in which the options were neutral about risk. We found that the surprise-sensitive utility model had the largest model evidence (Supplementary Figure S4A) and had a decisively higher protected exceedance probability (Supplementary Figure S4B). Each estimated parameter of the model is shown in Supplementary Figure S4C.

**FIGURE 4:**
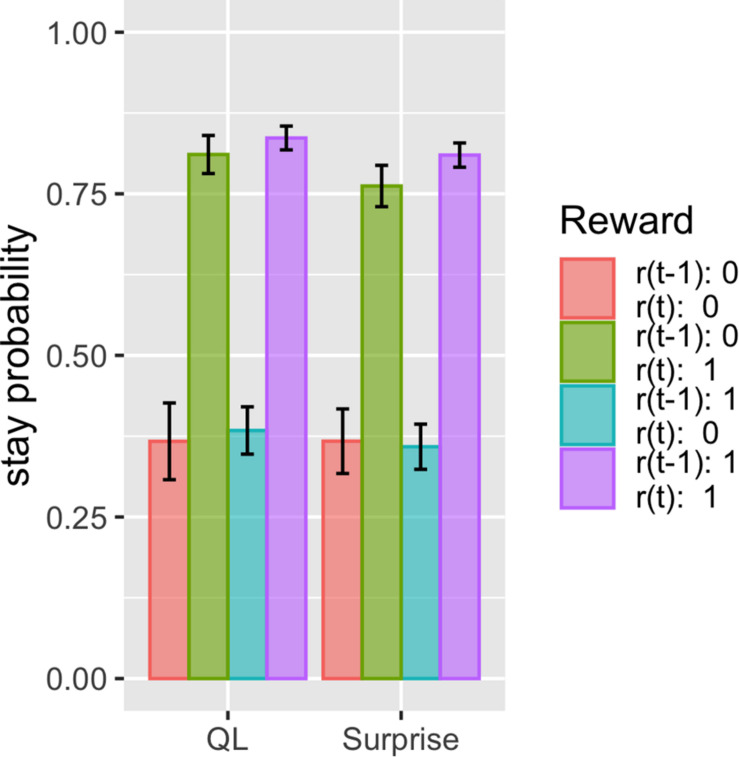
Effect of reward history on stay probability of the Q-learning model and surprise-sensitive utility model. Data are presented as the mean ± standard error. We found a smaller stay probability in proximate reward conditions [*r*(*t*) = 1], especially in the surprised reward condition [*r*(*t*) = 1, *r*(*t* − 1) = 0], in the surprise-sensitive utility model compared with the Q-learning model. QL, Q-learning model; surprise, surprise-sensitive utility model.

**FIGURE 5:**
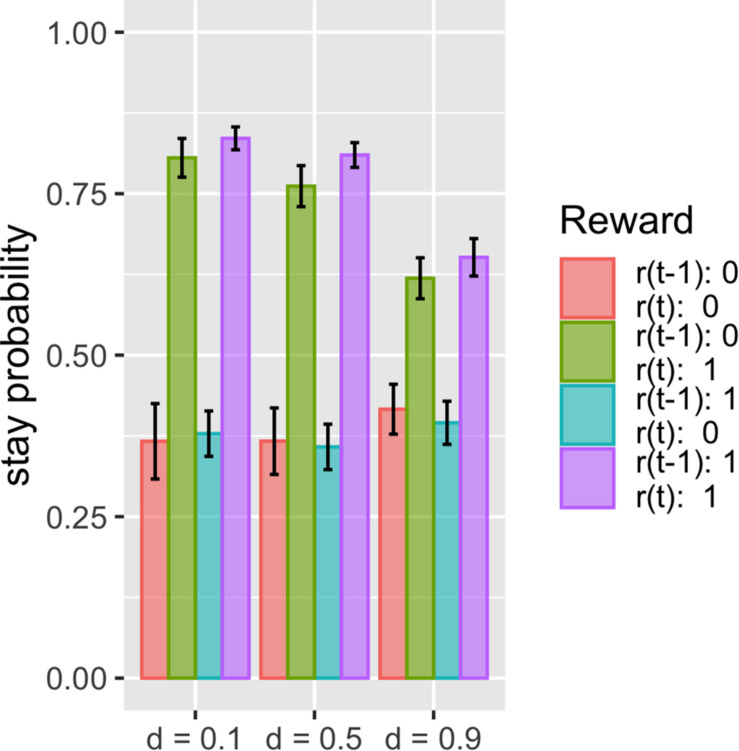
Stay probability and surprise decay rate. Data are presented as the mean ± standard error. We investigated how the surprise decay rate (*d*) affected the stay probability and found that as the parameter increased, the stay probability of proximate reward conditions [*r*(*t*) = 1] decreased.

The proposed model fits these datasets better than the other models, with the same number of free parameters. This suggests that there should be some difference in how the model predicts choice given the choice history and reward history. We thus investigated what kind of history dependence in the proposed model causes a difference in prediction from other models. To do this, we simulated the simple two-armed bandit task and compared the basic Q-learning model and surprise-sensitive utility model focusing on the relationship between stay probability and reward history. We found a smaller stay probability in proximate reward conditions [*r*(*t*) = 1; green and purple bars), especially in the surprised reward condition (*r*(*t*) = 1, *r*(*t* − 1) = 0; green bar], in the surprise-sensitive utility model compared with the Q-learning model (Figure 4). The difference in the decrease in stay probability on [*r*(t) = 1, *r*(t − 1) = 0] compared with [*r*(t) = 1, *r*(t − 1) = 1] was small, but this slight difference may account for the difference in the fit of models. We further investigated how the surprise decay rate (*d*) affected the stay probability and found that as the parameter increased, the stay probability of proximate reward conditions [*r*(*t*) = 1] decreased (Figure 5).

Finally, we studied the basic performance of the proposed model as a reinforcement learning algorithm (i.e., how the model successfully chooses the optimal choice). We conducted two additional simulations on a simple two-armed bandit task with different options of probability or outcome value. First, we simulated two safe options (reward probability is *p* = 0.9) with a small difference in reward magnitude (*r* = 1 or 0.8). As expected, the proposed model chose a larger reward option less frequently than the Q-learning model did (Supplementary Figure S5A). This is because the nearly uniform reduction in reward value by surprise for both options increased choice randomness (a similar effect as decreasing inverse temperature parameter, β). In contrast, when the difference in reward magnitude was large (*r* = 1 vs. *r* = 0.3), the proposed model chose the higher-reward option more frequently than the Q-learning model (Supplementary Figure S5B). This perhaps counterintuitive result is explained as follows. When the difference in reward magnitude was large, the model easily chooses the better option. This led to an imperfect action value update for the small-reward option. Thus, choosing a smaller reward option may easily generate a larger prediction error compared with the larger reward option, and thus, the action value of the lower valued option is more reduced by surprise. This results in the frequent choice of the large reward option.

## Discussion

In this study, we proposed the surprise-sensitive utility model in which prediction error works not only to update the prediction but also to decrease the outcome value. As a result of the simulation, we found that the surprise-sensitive utility model similarly explains the risk-averse choices to a previous model (Niv et al., 2012; Figure 1). We confirmed the superiority of the proposed model by model comparison using two actual experimental datasets (Figures 3A,B and Supplementary Figure S2). These findings suggest that surprise can affect subsequent decision making by reducing outcome value.

In the proposed model, the probability of choosing a sure option increased as the surprise decay rate (*d*) increased (Figure 2). This tendency increased as the learning rate (α) increased. This makes sense, as the larger surprise decay rate leads to a larger reduction in the value for the risky choice, making the agents avoid the choice. The learning rates determine the degree of the prediction update. Having large learning rates causes the agent to adjust their behavior quickly to avoid risky choices.

The proposed model fits these datasets better than the previous models, with the same or fewer free parameters. First, this model fits the dataset of Niv et al. (2012) and Goris et al. (2019) better than the risk-sensitive Q-learning model, which utilized separate learning rates for positive or negative prediction error, having asymmetric effects on changes in predictions. The result in which the proposed model fits these datasets better than the asymmetric learning rates model implies the importance of non-linear modulation of the evaluation of outcomes. At this point, the proposed model is similar to the utility model, but unlike the static modulation of the utility model, the modulation varies trial-by-trial in the proposed model. Because parameter estimates obtained from model fitting reflect not only the steady-state but also transient trial-by-trial behavior dynamics (Katahira, 2018), we believe that these differences between models may contribute to the fitting results.

This surprise sensitive utility model may explain the symptomatic behavior of psychiatric disorders, such as autism spectrum disorder (ASD). Our results showed that a decreased outcome value, a reward history that generates larger surprises, and a higher surprise decay rate all negatively affected an agent’s stay probability. These findings indicate that agents who are susceptible to surprise avoid making the same choice, even when the proximate outcome is rewarded. Individuals with ASD prefer repetitive behaviors with a perfect contingency that does not generate large surprises over social interaction, where contingency is non-perfect and surprise is more likely (Gergely, 2001; Van de Cruys et al., 2014; Northrup, 2017). Previous models cannot explain such preferential behaviors because, in these models, a positive prediction error leads to a preference for the chosen option. However, in the surprise-sensitive utility model, the value of the surprise outcome decreases, which can explain the tendency to avoid social interaction and prefer repetitive behaviors. Additionally, on average, individuals with ASD are known to have lower social motivation than neurotypical people (Chevallier et al., 2012; Bottini, 2018; Clements et al., 2018; Sumiya et al., 2020). However, at the individual level, individuals with ASD exhibit highly variable levels of social motivation (Sumiya et al., 2018). The surprise decay rate may be associated with these individual differences in social motivation within ASD. In the future, it may be useful to expand the use of this proposed model to investigate the differences in choice preference in individuals with ASD whose preference is influenced by the surprise.

## Conclusion

Previously, the effect of surprise on the value of an outcome and subsequent decision making was unclear. In this study, we proposed the surprise-sensitive utility model, a reinforcement learning model in which surprise decreases the outcome value. Comparing the proposed model with previous models, we found that the new model explains risk-averse choices as well as previous models. Risk-averse choices increased as the surprise-based modulation parameter of outcome value increased. The present findings suggest that surprise can affect subsequent decision making by reducing the outcome value. This proposed model can be the basis for a model linking the experimental data and the notion that surprise decreases outcome values.

## Data Availability Statement

Publicly available datasets were analyzed in this study. This data can be found here: https://nivlab.princeton.edu/data; https://osf.io/pkq3u.

## Ethics Statement

Ethical review and approval was not required for the study on human participants in accordance with the local legislation and institutional requirements. Written informed consent from the patients/participants or patients/participants legal guardian/next of kin was not required to participate in this study in accordance with the national legislation and the institutional requirements.

## Author Contributions

Both authors contributed to the conception and design of the study, wrote the manuscript, and contributed to manuscript revision, read and approved the submitted version. MS performed the statistical analysis.

## Conflict of Interest

The authors declare that the research was conducted in the absence of any commercial or financial relationships that could be construed as a potential conflict of interest.

## Footnotes

1 https://nivlab.princeton.edu/data

2 https://osf.io/pkq3u

## Supplementary Material

FIGURE S1 Probability of choosing a sure option for each subject. We found that behavioral performance on our proposed model is more similar to the real data than other models for some subjects, especially those whose probability of choosing a sure option is high. QL, Q-learning model; utility, utility model; RSQL, risk-sensitive Q-learning model; surprise, surprise-sensitive utility model.

FIGURE S2 Plot and second-order polynomial fit for the probability of choosing a sure option depending on the surprise decay rate (−1≤*d*≤1) and learning rate. When a surprise decay rate (*d*) is positive, risk aversion increases as the surprise decay rate (*d*) increases. However, when a surprise decay rate (*d*) is negative, risk aversion decreases as *d* decreases.

FIGURE S3 Estimated parameters of the surprise-sensitive utility model in the Niv et al. (2012) dataset.

FIGURE S4 The results of the analysis on the dataset of Goris et al. (2019). **(A)** Model evidence for each model: data are presented as the mean (bars) and data value (plots and lines) per subject, **(B)** Bayesian model selection, **(C)** estimated parameters of the surprise-sensitive utility model. We found that the surprise-sensitive utility model had the largest value and had a decisively higher protected exceedance probability. QL, Q-learning model; utility, utility model; RSQL, risk-sensitive Q-learning model; surprise, surprise-sensitive utility model.

FIGURE S5 Numbers for choosing each option of the Q-learning model and surprise-sensitive utility model. Data are presented as the mean ± standard error. **(A)** We compared two safe options (*p* = 0.9) with a small value difference (*r* = 1 or 0.8) and found that agents chose higher valued options less in the proposed model than in the Q-learning model. **(B)** We compared two safe options (*p* = 0.9) with higher values (*r* = 1) and lower values (*r* = 0.3) and found that agents chose the higher-value option in the proposed model more than the Q-learning model. QL, Q-learning model; surprise, surprise-sensitive utility model.

TABLE S1 Model specification.
