# Supplementary figures and images for: Surprise Acts as a Reducer of Outcome Value in Human Reinforcement Learning
Source: Front Neurosci. 2020 Sep 8;14:852. doi: 10.3389/fnins.2020.00852 (PMC7506125; doi:10.3389/fnins.2020.00852)

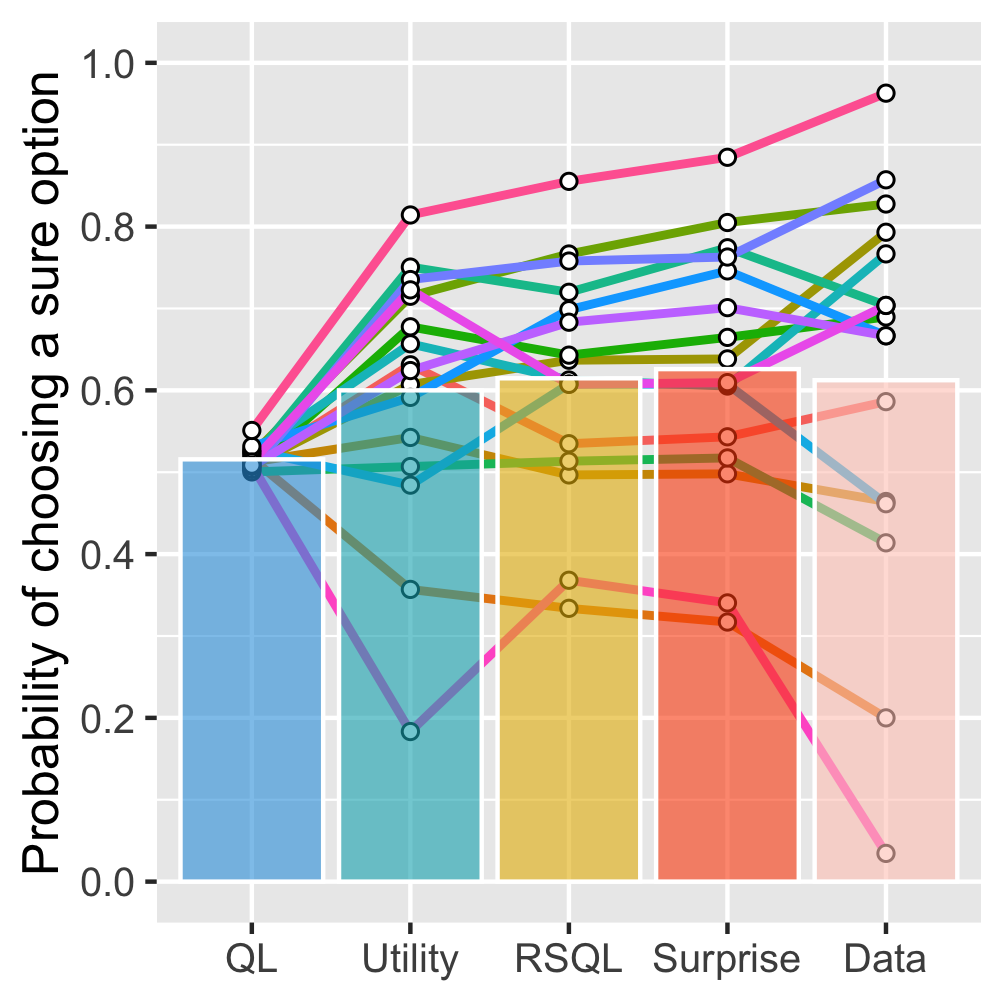

Supplement: FIGURE S1 — Probability of choosing a sure option for each subject. We found that behavioral performance on our proposed model is more similar to the real data than other models for some subjects, especially those whose probability of choosing a sure option is high. QL, Q-learning model; utility, utility model; RSQL, risk-sensitive Q-learning model; surprise, surprise-sensitive utility model. [file Image_1.TIFF]

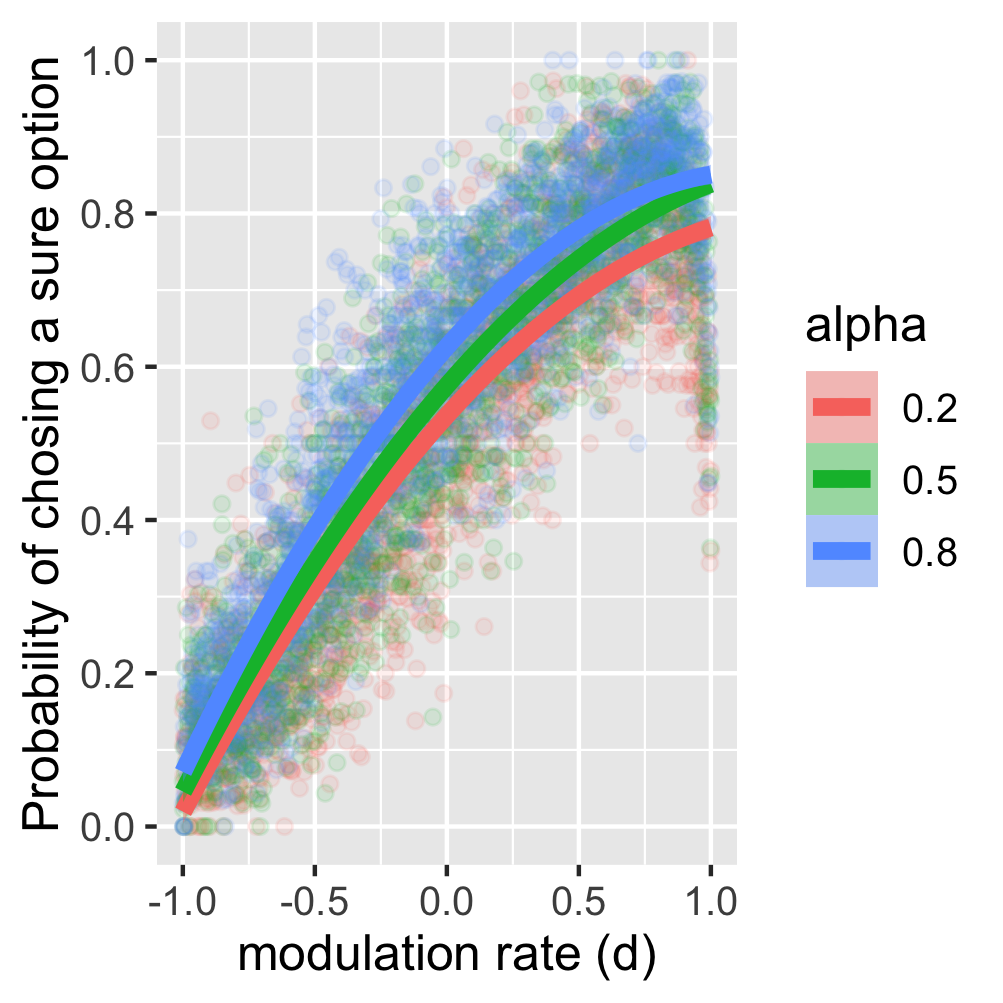

Supplement: FIGURE S2 — Plot and second-order polynomial fit for the probability of choosing a sure option depending on the surprise decay rate (−1≤d≤1) and learning rate. When a surprise decay rate (d) is positive, risk aversion increases as the surprise decay rate (d) increases. However, when a surprise decay rate (d) is negative, risk aversion decreases as d decreases. [file Image_2.TIFF]

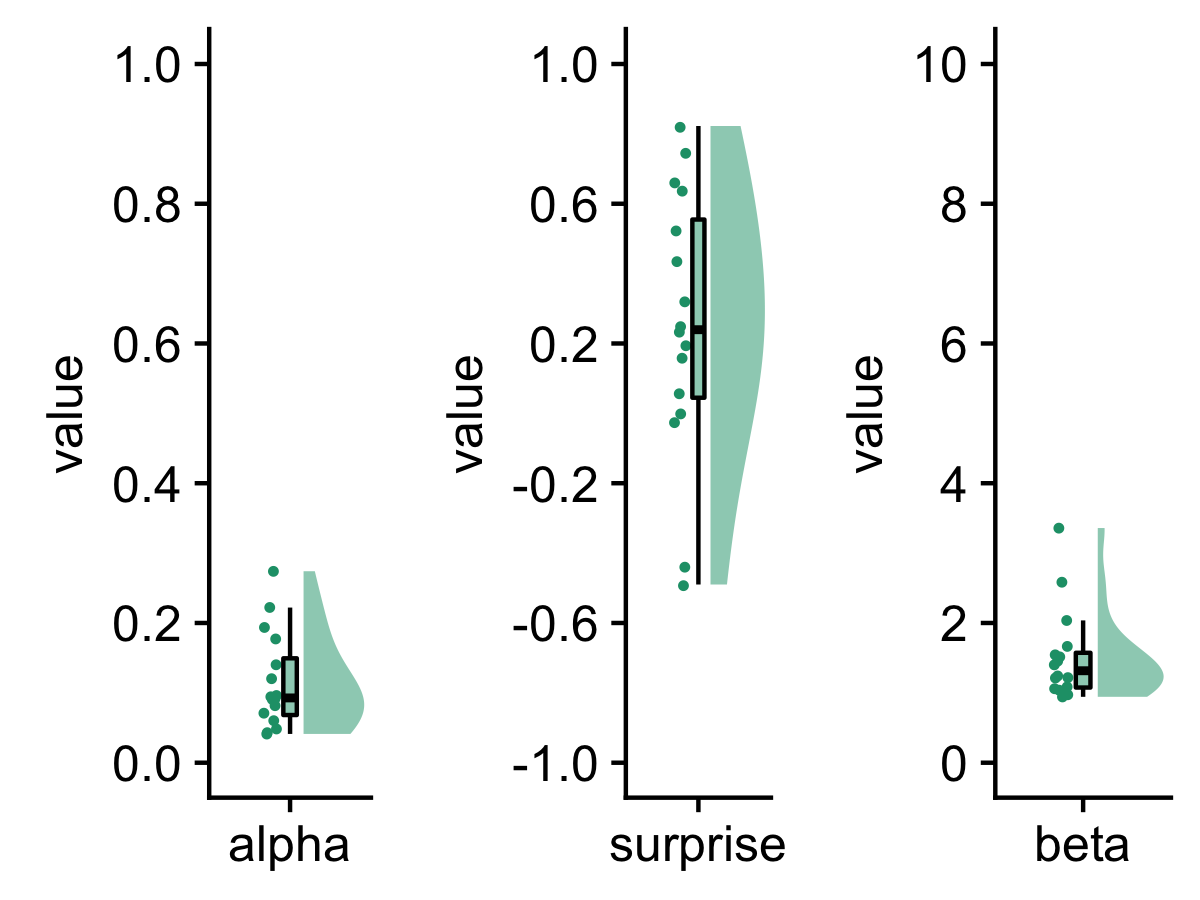

Supplement: FIGURE S3 — Estimated parameters of the surprise-sensitive utility model in the Niv et al. (2012) dataset. [file Image_3.TIFF]

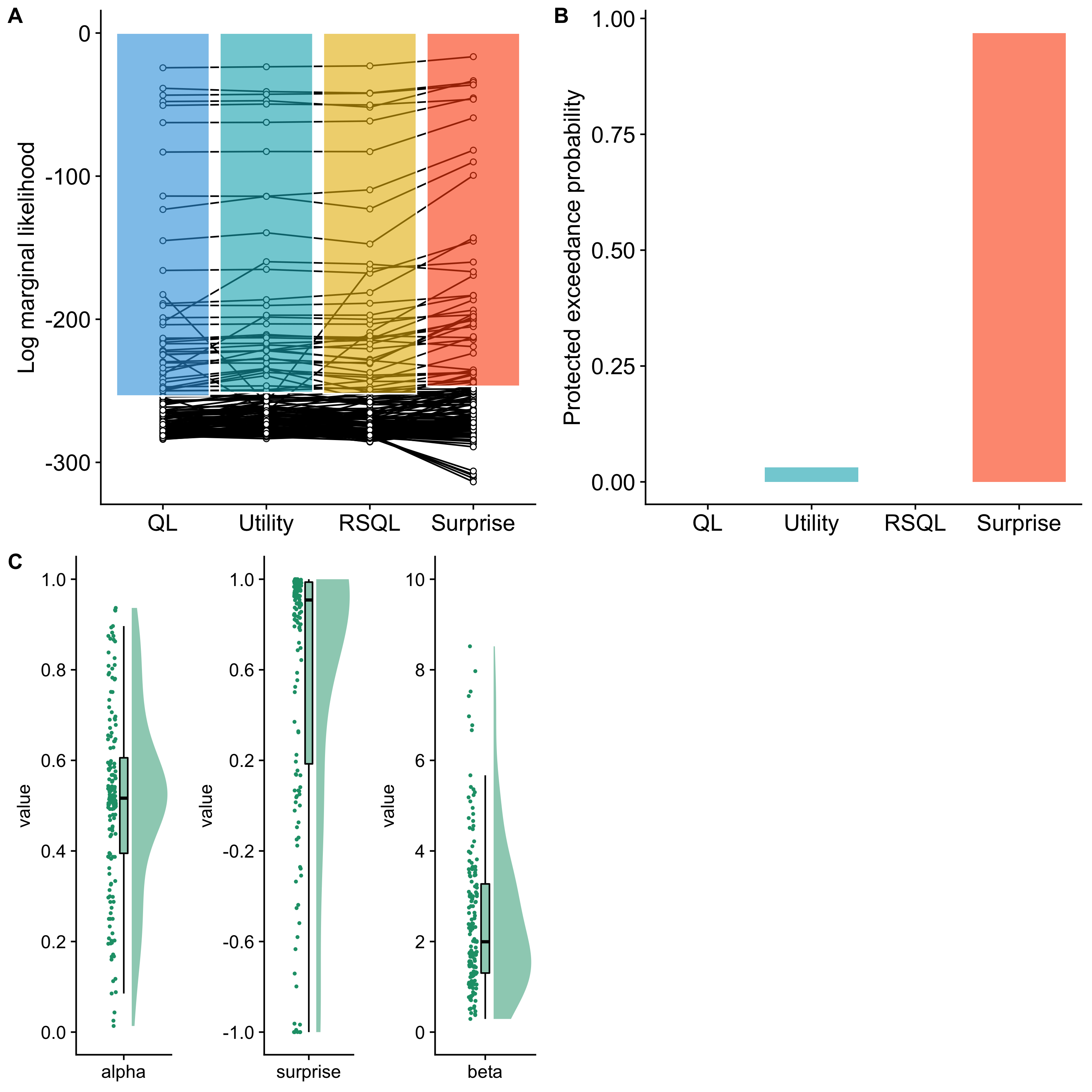

Supplement: FIGURE S4 — The results of the analysis on the dataset of Goris et al. (2019). (A) Model evidence for each model: data are presented as the mean (bars) and data value (plots and lines) per subject, (B) Bayesian model selection, (C) estimated parameters of the surprise-sensitive utility model. We found that the surprise-sensitive utility model had the largest value and had a decisively higher protected exceedance probability. QL, Q-learning model; utility, utility model; RSQL, risk-sensitive Q-learning model; surprise, surprise-sensitive utility model. [file Image_4.PNG]

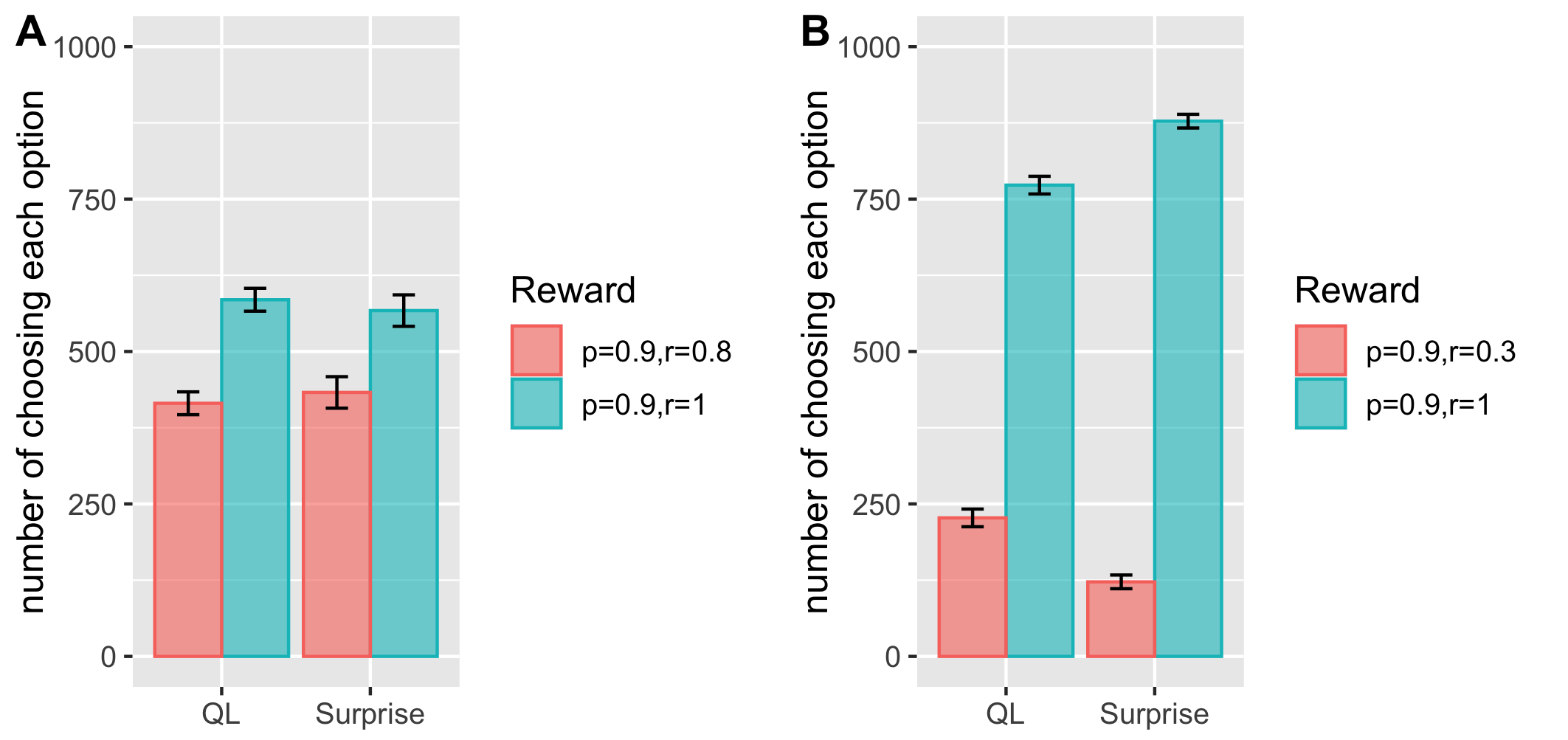

Supplement: FIGURE S5 — Numbers for choosing each option of the Q-learning model and surprise-sensitive utility model. Data are presented as the mean ± standard error. (A) We compared two safe options (p = 0.9) with a small value difference (r = 1 or 0.8) and found that agents chose higher valued options less in the proposed model than in the Q-learning model. (B) We compared two safe options (p = 0.9) with higher values (r = 1) and lower values (r = 0.3) and found that agents chose the higher-value option in the proposed model more than the Q-learning model. QL, Q-learning model; surprise, surprise-sensitive utility model. [file Image_5.TIFF]
