# Supplementary material for: Surprise Acts as a Reducer of Outcome Value in Human Reinforcement Learning
Source: Front Neurosci. 2020 Sep 8;14:852. doi: 10.3389/fnins.2020.00852 (PMC7506125; doi:10.3389/fnins.2020.00852)
Supplement: TABLE S1 — Model specification. [file Table_1.pdf]

S1 table: Model Specification

	N of param	α _P	α	α _N	β	u	d
QL	2		○		○		
Utility	3		○		○	○	
RSQL	3	○		○	○		
Surprise	3		○		○		○

Name of models (QL: Q-learning model, Utility: utility model, RSQL: risk-sensitive Q-learning model, Surprise: surprise sensitive utility model).

N of param: number of free parameters
